# Guided Healing of Damaged Microelectrodes via Electrokinetic Assembly of Conductive Carbon Nanotube Bridges

**DOI:** 10.3390/mi12040405

**Published:** 2021-04-06

**Authors:** Tuo Zhou, Matthew Michaels, Lawrence Kulinsky

**Affiliations:** 1 Department of Mechanical and Aerospace Engineering, University of California, Irvine, 5200 Engineering Hall, Irvine, CA 92627-2700, USA; tuoz3@uci.edu; 2 Department of Materials Science and Engineering, University of California, Irvine, 544 Engineering Tower, Irvine, CA 92627-2700, USA; mmichae3@uci.edu

**Keywords:** electrokinetic healing of electrodes, CNT, dielectrophoresis, electrokinetic assembly

## Abstract

The subject of healing and repair of damaged microelectrodes has become of particular interest as the use of integrated circuits, energy storage technologies, and sensors within modern devices has increased. As the dimensions of the electrodes shrink together with miniaturization of all the elements in modern electronic devices, there is a greater risk of mechanical-, thermal-, or chemical-induced fracture of the electrodes. In this research, a novel method of electrode healing using electrokinetically assembled carbon nanotube (CNT) bridges is presented. Utilizing the previously described step-wise CNT deposition process, conductive bridges were assembled across ever-larger electrode gaps, with the width of electrode gaps ranging from 20 microns to well over 170 microns. This work represents a significant milestone since the longest electrically conductive CNT bridge previously reported had a length of 75 microns. To secure the created conductive CNT bridges, they are fixed with a layer of electrodeposited polypyrrole (a conductive polymer). The resistance of the resulting CNT bridges, and its dependence on the size of the electrode gap, is evaluated and explained. Connecting electrodes via conductive CNT bridges can find many applications from nanoelectronics to neuroscience and tissue engineering.

## 1. Introduction

The durability and longevity of electronic components, including electrically conductive traces and interconnections, are becoming increasingly more important due to the ever-decreasing scale of electronic devices [1,2,3,4]. However, as new developments in micro and nano fabrication enable these components to become dramatically smaller in size and applications of microdevices have expanded, electrodes are exposed to greater risk of mechanical, thermal, and chemically induced damage [1,2,3,5]. Therefore, the subject of electrode repair gains new importance as it brings concurrent benefits of longer lifetime, better device reliability, and reduced waste [4,5,6,7].

Much of the previous research in the field of microelectrode repair has focused on encapsulation in self-restorative polymers which can repair cracks and discontinuities that develop in the electrode. For example, in a 2013 article, Wang et al. successfully demonstrated the use of a hydrogen-bond-directed self-healing polymer binder as a self-healing mechanism for silicon-based anodes in lithium ion batteries [8]. The polymer-based solution was used to repair electrodes fractured due to the cyclic lithiation/delithiation electrochemical process and the attendant significant volumetric expansion and contraction of the silicon electrodes [8].

Another method to achieve microelectrode repair via encapsulation was presented by Sim et al. in which highly conductive silver nanowires are encapsulated in liquid crystal graphene oxide (LCGO). The use of LCGO, as compared to more traditional polymers and hydrogels, offered better restoration of electrical conductivity and of tensile strength [9].

Healing of microelectrodes by sputtering metals on top of polymetric microfibers has also been demonstrated. In Wang et al.’s 2018 study, a gold coating was sputtered onto a single-layer microfibrous polycaprolactone network. Under applied strain, cracks developed in the conductive gold coating while the underlying polymer system is elongated but not fractured. The polymer fibers were successfully shrunk through heat treatment via the application of steam, while the shrinkage of the underlying polymer network brought fractured gold coating back into contact, restoring conductivity [10].

Other research into fabrication of more durable and reliable electrically-conductive traces has focused on the development of flexible liquid-metal electrodes. In 2013, Liu et al. proposed a method to direct print metallic films of GaInSn alloy for use as electrodes within dielectric elastomer actuators. The liquid metal electrodes demonstrated significant compliance under applied stress as well as relatively stable resistance with increasing strain. Furthermore, this liquid metal electrode possessed two-dimensional self-healing capability to repair gaps formed within the electrode, adding to its reliability [11]. Further research into the development of flexible electrodes includes the use of biomimetic elastomeric micropore film to increase the tensile limit and electrical stability of common conductive substrates and metals [12].

The approach to guided repair of microelectrodes presented in this study focuses on the use of carbon nanotubes (CNTs) known for their impressive electrical and mechanical properties [13]. Since their introduction, CNTs have become ubiquitous components in a variety of microdevices including sensors, batteries, and integrated circuits [14,15].

Many assembly techniques have been developed to manipulate and incorporate CNTs into materials and devices [16,17,18]. Our research focuses on electrokinetic assembly that employs electrical forces to manipulate small particles to create micro and nanoscale structures. Specifically, dielectrophoretic (DEP) force is utilized to manipulate and arrange the CNTs. The DEP force acts on micro and nanoparticulate in a non-uniform electric field [19,20]. In our studies we use an externally applied alternating current (AC) electric field [20]. Microparticles subjected to an electric field experience polarization. The resulting dipole moment of the particle aligns with the electric field lines and, depending on the applied frequency and physical properties of particles and media, this applied force can either attract or repel particles from the electrodes [19,20]. The magnitude of the particle polarization and resulting DEP force applied to an elongated particle is described in Equation (1) [21,22,23,24,25,26].

(1) $$F_{DEP}=\frac{\pi r^{2}l}{6}\varepsilon_{m}Re\left[f_{m}\right]\nabla E_{rms}^{2},$$

where l and *r* are the length and radius of the particle, respectively; $\varepsilon_{m}$ is the permittivity of the medium;$\;E_{rms}$ is the root means square of the electric field; and $Re\left[f_{m}\right]$ is the real part of the Clausius-Mossotti (CM) factor, $f_{m}$, described in Equation (2):

(2) $$f_{m}=\frac{\varepsilon_{n}^{*}-\varepsilon_{m}^{*}}{\left(\varepsilon_{n}^{*}-\varepsilon_{m}^{*}\right)A_{L}+\varepsilon_{m}^{*}},$$

where $A_{L}$ is the depolarization factor, and $\varepsilon_{n}^{*}$ and $\varepsilon_{m}^{*}$ are the complex permittivities of particle and medium, respectively, as defined by Equation (3):

(3) $$\varepsilon^{*}=\varepsilon -j\frac{\sigma }{\omega },$$

where ε and σ are the permittivity and conductivity, respectively, and ω is the frequency of the applied electric field [21,22,23,24,25,26].

When the real part of the CM factor is positive, the particles are attracted to electrodes; alternatively, when the real part of the CM factor is negative, the particles are repelled from the electrodes [21,22,23,24,25,26].

In addition to the use of DEP force for electrokinetic assembly [21,22,23,24,25,26], this research utilizes the step-wise CNT deposition process previously reported by Zhou et al. [18]. This method involves iterative deposition of small droplets of CNTs suspended in isopropyl alcohol (IPA) and allowing the IPA to evaporate between depositions. It was hypothesized that during the evaporation step, capillary forces of the contracting liquid meniscus bring neighboring chains of CNTs into tighter contact, thus reducing the CNT-to-CNT contact resistance. This novel process is critical in enabling the construction of conductive CNT bridges across much wider electrode gaps than previously reported. The step-wise CNT deposition was successfully used to create a conductive CNT bridge over 75 micron long [18]. However, the electrode geometry utilized in that study (a straight microelectrode surrounded by a horseshoe-shaped electrode) produced a non-uniform field conducive to strong DEP forces and the question remained about the applicability of this approach to the conventional straight electrodes with gap in between or to the creation of the conductive CNT bridge to repair the broken electrodes. Present research conclusively demonstrates the validity and feasibility of the step-wise DEP deposition of conductive CNT bridges to heal the broken conventional microelectrodes.

The combination of DEP electrokinetic assembly paired with step-wise deposition of CNTs demonstrated in this research creates a process which is self-reinforcing, i.e., as the process progresses, the magnitude of the DEP force increases (as illustrated by the COMSOL simulation below). Additionally, the effective resistance of the contact junctions between successive CNT chains is evaluated for CNT bridges of various lengths.

Fabrication of long conductive CNT bridges, besides their application in micro- and nano-electronics will find widespread application in many other fields, including biotechnology and tissue engineering. For example, research conducted by Hallstrom et al. has demonstrated regenerative neural growth of axons along conductive nano-wire arrays [27]. Such technology may help treat neurological damage in patients, including spinal cord injuries [27,28,29].

## 2. Materials and Methods

### 2.1. Fabrication of Carbon Electrodes

**Figure 1:**
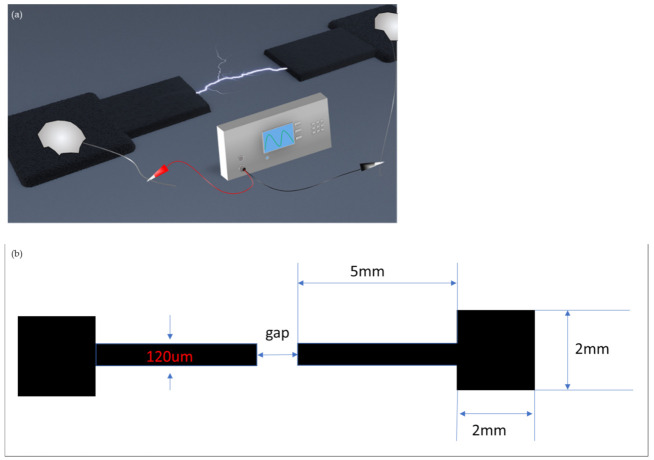
(**a**) Carbon nanotube (CNT) electrokinetic assembly experimental setup design. (**b**) Dimensions of the carbon electrodes.

Various sets of carbon microelectrodes with electrode gaps of 20, 30, and 40 microns were produced by initially photopatterning SU-8 resist via a standard lithographic process and then pyrolyzing a SU-8 precursor in a reductive nitrogen environment of the furnace that converts organic resist into glassy carbon material. Figure 1b presents the geometry of the microelectrodes: the width of the electrode fingers is 120 μm, while the length of the opposing electrode segments are 5 mm each. These microelectrodes terminate in a square contact pad with the sides of 2 mm wide. Additionally, a set of electrodes was produced that did not have any gaps and after these “undamaged” electrodes were produced, we scratched these electrodes, creating a realistic break that was wider than 170 microns (see Section 3.3 below). The details of the fabrication process of the carbon electrodes are given below.

A 4” silicon wafer covered with a 1 μm oxide layer (University Wafer, Boston, MA, USA) was spin-coated with SU-8 2015 photoresist (Kayaku Inc., Westborough, MA, USA) using a Laurell photoresist spinner (Laurell Technologies, North Wales, PA, USA) at an initial speed of 500 rpm for 10 s, followed by 4000 rpm spin for 30 s. A hot plate was used for soft-baking at 95 °C for 2 min. The subsequent exposure step was performed using a photomask (CadArt, Bandon, OR, USA) and ultraviolet (UV) light at an energy intensity of 10 mW/cm^2^ for 10 s in a Karl Suss MA56 Mask Aligner (Karl Suss, Garching, Germany). The wafer was then post-baked on the same hot plate at 95 °C for 4 min. The undeveloped resist was removed with SU-8 developer (Kayaku Inc., Westborough, MA, USA). The remaining developed resist layer underwent hot-baking at 150 °C for 20 min. After hot-baking, the resist layer was carbonized inside a pyrolysis furnace (Thermo Fisher Scientific, Waltham, MA, USA), within a nitrogen-rich environment. The pyrolysis process was initiated at 25 °C for two hours, followed by a 69 min ramp to 300 °C, where the temperature was maintained for one hour before a subsequent 90 min ramp to 900 °C, where the temperature was maintained again for one hour before being allowed to naturally cool to room temperature overnight. Finally, 34-gauge buss wire (Guasti Wire and Cable, Ontario, Canada) was soldered onto the contact pads of the electrode using Indium solder.

### 2.2. Preparation of Carbon Nanotube Suspension

The CNT suspension was prepared by adding 0.005 g of multi-wall CNT (MWCNT) powder (Sigma-Aldrich, St. Louis, MO, USA) into 15-mL of isopropyl alcohol (IPA). The solution underwent centrifugation in an Eppendorf 5702 Centrifuge (Eppendorf AG, Hamburg, Germany) at 3000 rpm for 4 h. The large agglomerates of CNTs had precipitated and the supernatant containing low concentration of suspended CNTs was pipetted out.

### 2.3. Experimental Setup and Deposition of Carbon Nanotube Suspension

The chip containing the carbon electrode array was placed under an optical microscope (Nixon Eclipse, Minato, Japan) which was connected to a cMOS video camera (SPOT Imaging, Sterling Heights, MI, USA). The buss wires soldered onto the carbon electrode were connected to a function generator (Stanford Research Systems, Sunnyvale, CA, USA) which was set up to apply AC bias at a frequency of 100 kHz and the constant peak-to-peak voltage (Vpp) of 4 V. The experimental setup is depicted in the diagram in Figure 1a.

A 0.5 μL of the CNT suspension was pipetted (Labnet, Edison, NJ, USA) on top of the electrode gap. The droplet of CNT suspension was allowed to fully evaporate, which could be visually confirmed under the optical microscope. Once fully evaporated, the next droplet of CNT suspension was deposited. This step-wise deposition continued until the CNT bridge could be seen spanning the entirety of the gap. Once the conductive CNT bridge was completed, the wires were disconnected from the function generator and a resistance measurement across the electrode was taken using a multimeter (Wavetek, San Diego, CA, USA). In order to assess the efficacy of the healing process, resistance across a reference electrode containing no gap was also measured. Finally, high magnification images of the CNT bridges were taken using a Magellan 400 XHR scanning electron microscope (FEI, Hillsboro, OR, USA).

### 2.4. Preperation of Pyrrole Solution

A 0.1M pyrrole monomers (Sigma-Aldrich, St. Louis, MO, USA) and 0.1 M NaDBS (sodium dodecylbenzenesulfonate) (Sigma-Aldrich, St. Louis, MO, USA) were mixed with 100 mL of deionized water and stirred for 20 min at room temperature.

### 2.5. Polypyrrole Deposition

In order to permanently affix the CNT bridge to the electrodes and the substrate, the layer of electroactive polymer polypyrrole (PPy) was electrodeposited atop of the CNT bridge. The pyrrole solution was pipetted (Labnet, Edison, NJ, USA) over the deposited CNT bridge while a 0.9 V direct current (DC) bias was applied by the function generator (Stanford Research Systems, Sunnyvale, CA, USA). Two different methods were evaluated for the deposition of PPy. In one approach, the DC bias was applied between the working electrode (one of the microelectrodes) and the counter-electrode (the microelectrode across the gap) and the deposition proceeded from one microelectrode and across the CNT bridge until the PPy layer reached the other microelectrode on the other side of the gap. In the alternative deposition technique, the initial deposition started as previously described, but once the PPy layer reached the approximate position of half-way through the CNT bridge, the electrodes for DC bias were switched and the PPy deposition will start from the opposite side of the bridge and the deposition will be finished when the PPy layers will merge. The differences between these two PPy deposition methods are evaluated in Section 3.4 below.

### 2.6. Stress-Testing of Carbon Nanotube (CNT) Bridges

Once fabricated, samples with the CNT bridges across 30 μm gaps, with and without PPy coat, were tested to determine how resilient they were to several types of environmental stress, including the forceful blasts of compressed nitrogen gas, placing the samples under the running water stream, and exposing the sample to thermal cycling.

Each sample was subjected to 10 rounds of thermal cycling during which they were brought to 200 °C using a hot plate and then cooled to 3 °C using an ice pack. There was no resident time at the top or bottom temperature—once the temperature was reached, the sample was immediately moved to the other temperature extreme. Next, each sample were placed under a stream DI water running with a volumetric flow rate of 0.06 L per minute (LPM) for a total of 30 s. Finally, each sample was blown with nitrogen gas at 88 pounds of force per square inch (psi) for 30 s. After each test, the resistance of the electrode was measured.

### 2.7. Finite Element Analysis of Electric Field Magnitude

A simulation was conducted in the Comsol Multiphysics Software package (v. 5.2) (Comsol, Burlington, MA, USA) to analyze the strength of the electric field strength resulting from applied AC bias across electrode gaps of various lengths. The square of the electric field strength was also analysed. The fine geometry-controlled triangular mesh containing more than 18,000 elements was implemented. The model used a glassy carbon electrode material and the surrounding IPA medium with permittivities of 108⋅ε_0_ and 18.23⋅ε_0_, respectively. A built-in linear solver was utilized to calculate the solution of the governing (Laplace) system of equations. The boundary conditions were set to be 0 for one electrode and 4V for another electrode.

## 3. Results and Discussion

### 3.1. CNT Bridges across 20, 30 and 40 μm Electrode Gaps

#### 3.1.1. Assembly of CNT Bridges

**Figure 2:**
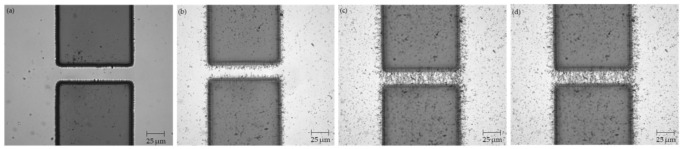
Electrodes with 20 μm gap (**a**) before CNT deposition; (**b**) after 2 μL of CNT suspension; (**c**) after 4 μL of CNT suspension; (**d**) after 5 μL of CNT suspension.

**Figure 3:**
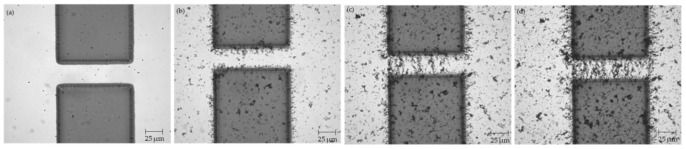
Electrodes with 30 μm gap (**a**) before CNT deposition; (**b**) after 3 μL of CNT suspension; (**c**) after 7 μL of CNT suspension; (**d**) after 10 μL of CNT suspension.

**Figure 4:**
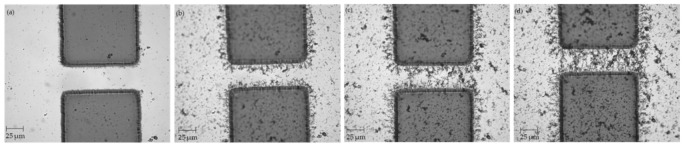
Electrodes with 40 μm gap (**a**) before CNT deposition; (**b**) after 5 μL of CNT suspension; (**c**) after 7 μL of CNT suspension; (**d**) after 10 μL of CNT suspension.

Using the procedure outlined in Section 2.3 above, CNT bridges were successfully assembled across the 20, 30, and 40 μm electrode gaps. Three samples were manufactured and tested for each type of electrode. Images were taken throughout the CNT deposition process using the optical microscope equipped with the cMOS camera and are presented in Figure 2, Figure 3, and Figure 4 below.

Table 1 below summarizes the resistance across the electrode with 30 μm gap after every deposition of every two 0.5 μL drops of CNT suspension.

**Figure 5:**
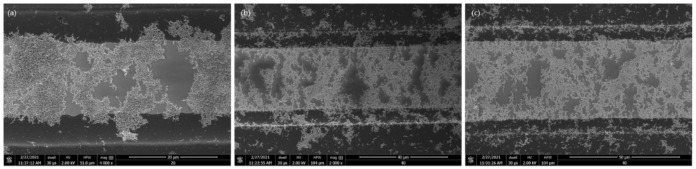
Scanning electron microscope (SEM) pictures of the CNT bridges formed across (**a**) 20 μm electrode gap (**b**) 30 μm electrode gap (**c**) 40 μm electrode gap.

As expected, the amount of CNT suspension required to form a complete conductive bridge increased as the electrode gaps become wider. The resistance across the electrode decreased exponentially with the increased number of the deposited CNT suspension droplets. Scanning Electron Microscopy (SEM) images in Figure 5 illustrate the morphology of the CNT bridges.

#### 3.1.2. Resistance of CNT Bridges

**Figure 6:**
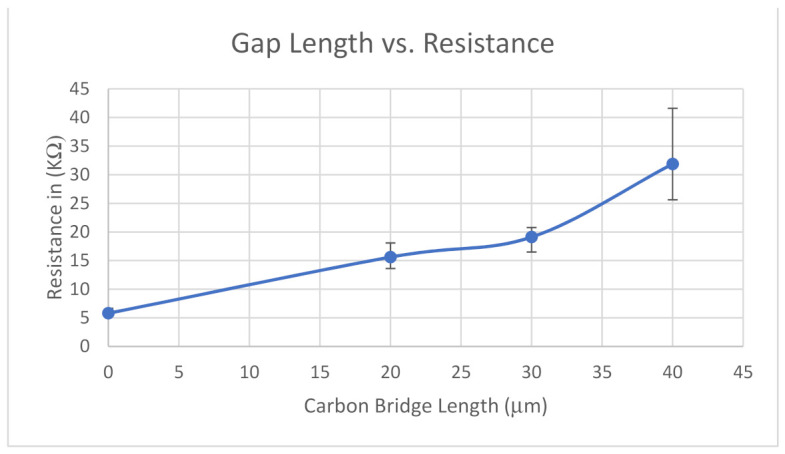
Plot of the measured resistance of the healed electrodes as function of CNT bridge length.

Resistance measurements taken across the healed electrodes are summarized in Table 2 below. Additionally, these resistance measurements are plotted as a function of CNT bridge length in Figure 6.

The graph in Figure 6 demonstrates that the resistance across the healed electrode increases with CNT bridge length. Previous research has concluded the primary contribution to the resistance across a CNT bridge is the resistance arising at the CNT-to-CNT contact junctions [30,31]. The results presented here suggest that the contact resistance grows as the length of the CNT bridge increases, since the increase in resistance is non-linear.

Based on the comparison of the resistance of undamaged (reference) electrodes and the healed electrodes (see Table 1) we can conclude that the electrokinetically assembled CNT bridges restored conductivity to the electrodes, but we could see a significant decrease in the repaired electrodes’ conductivity. The healed electrodes had resistance that was 169%, 229%, and 449% higher than the original resistance for the 20, 30, and 40 μm gap electrodes, respectively.

### 3.2. Electric Field Strength Simulation

**Figure 7:**
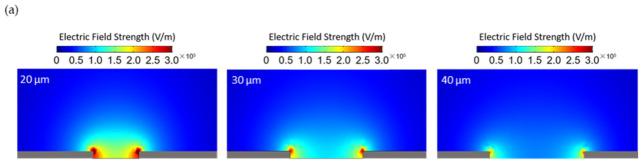

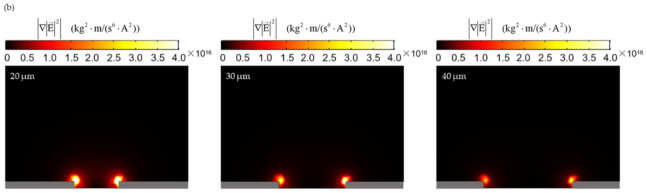
Simulation across electrode gap lengths of (**a**) the electric field strength (**b**) the gradient of the square of the electric field strength.

A simulation of electric field strength across varying electrode gap lengths was conducted using the multiphysics Comsol framework. As shown in Figure 7 below, the strength of the electric field is inversely proportional to the distance across which it is applied. This is an expected result according to Coulomb’s law describing the magnitude of on electric field [32].

This result indicates that as a conductive bridge is assembled and the distance between the opposing ends of the growing bridge spans become smaller, the applied electric field increases with the attendant increase in the DEP force (see Equation (1)) instrumental for the CNT bridge assembly. This self-reinforcing process is important for offset higher contact resistance of the CNT-to-CNT network away from the electrodes.

### 3.3. CNT Bridge across the Fractured Electrode

**Figure 8:**
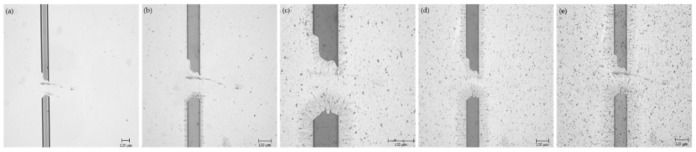
Fractured electrode (**a**) before CNT deposition; (**b**) after deposition of 7 μL of CNT suspension; (**c**) after 15.5 μL of CNT suspension; (**d**) after 19 μL of CNT suspension; and (**e**) after 23 μL of CNT suspension.

The same process used to assemble the CNT bridge across pre-manufactured electrode gaps was successfully applied to an electrode that was mechanically fractured using a sharp glass tip. The gap resulting from the fracture was expectedly non-uniform. At its smallest, the gap was over 170 μm wide and the largest gap was over 310 μm wide. Once broken, it was confirmed using a multimeter that the electrode had infinite resistance. After application of 23 μL of CNT suspension (in increments of 0.5 μL) per the method detailed in Section 2.3, a complete CNT bridge was formed across the gap as shown in Figure 8.

The resistance across the healed electrode was measured to be 239.6 kΩ representing a 4031% increase in resistance across the electrode. While the magnitude of this increase is large, these results serve as a successful proof of concept that the CNT bridge can be assembled across large (over 150 μm wide) gaps and restore electrical conductivity to an electrode using the electrokinetic assembly process described above.

### 3.4. Deposition of Polypyrrole

Using 0.9 V DC bias, PPy was successfully deposited across the CNT bridge to permanently affix it to the electrodes. Both methods for PPy deposition, as detailed in Section 2.5 were capable of covering the CNT bridge to secure it to the substrate.

**Figure 9:**
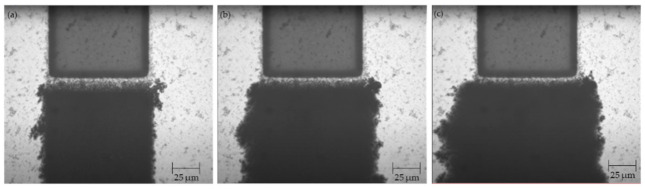
Results of polymer polypyrrole (PPy) deposition over the CNT bridge spanning 20 μm electrode gap without switching the polarity of 0.9 Vpp DC bias captured at approximately (**a**) 25% completion (**b**) 50% completion (**c**) 100% completion.

In the first PPy deposition approach the DC bias was used without changing the polarity during the deposition process (see Section 2.5 above). The results of this PPy deposition methodology are depicted in Figure 9. One notable feature of this PPy deposition method is the lateral growth of PPy from the side walls on the positive side of the electrode. As PPy grew towards the opposite electrode, it also grew laterally to each side of the electrode.

**Figure 10:**
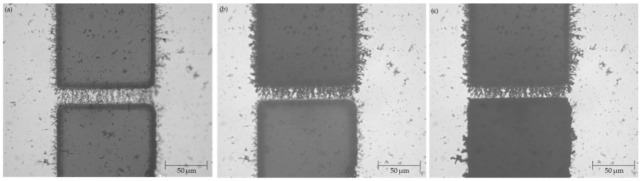
Results of PPy deposition over the CNT bridge spanning 20 μm electrode gap (**a**) prior to PPy application; (**b**) after the PPy deposition from the side of the top electrode reached roughly half-way through the CNT bridge and the polarity of the 0.9 Vpp DC bias was switched; (**c**) completion of PPy deposition over the CNT bridge.

**Figure 11:**
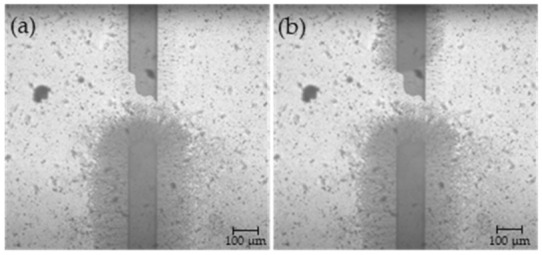
Results of the PPy deposition onto the CNT bridge of the healed electrode that contained a wide fracture (**a**) after the initial PPy deposition from the bottom electrode at 0.9 Vpp DC bias; (**b**) after the polarity of the DC bias was reversed.

In the second PPy deposition methodology where the polarity of the DC bias was switched once the PPy coat reached halfway through the electrode gap, there was smaller lateral growth of PPy compared to the first PPy deposition technique (Figure 10). Therefore, depending on the intended application and desire to have larger or smaller lateral PPy coverage, either the first or second PPy deposition technique can be utilized. PPy was also deposited onto the repaired electrode that had an initial fracture as demonstrated in Figure 11.

### 3.5. Results of the Stress Testing of the Formed CNT Bridges

#### 3.5.1. Thermal Cycling

Resistance measurements taken before and after 10 rounds of thermal cycling between 200 °C and 3 °C are summarized in Table 3 below.

The CNT bridge without PPy experienced a 19% decrease in resistance as a result of thermal cycling. This is likely due to the additional evaporation that occurs as the sample is heated and the bridge expands. This additional evaporation under the influence of the surface tension forces of the shrinking liquid meniscus [18] pulls the CNTs closer together, thus reducing the CNT-to-CNT contact junction resistance. A circumstantial evidence for that suggested mechanism of the decrease in CNT-to-CNT contact resistance is offered by the fact that when the CNT bridge is covered by the PPy layer that impedes evaporation, the overall resistance did not decrease, but actually increased by 17% as a result of thermal cycling.

#### 3.5.2. Placing the Healed Microelectrodes under Running Water

Resistance measurements before and after placing the samples for 30 s under a stream of deionised water (DI) with the volumetric flow rate of 0.06 LPM are summarized in Table 4 below. We can conclude that no significant increase in resistance was observed.

#### 3.5.3. Exposure of the Healed Microelectrodes to Blasts of Compressed Nitrogen Gas

Resistance measurements before and after blowing nitrogen gas at 88 psi for 30 s are summarized in Table 5 below.

There was no observed increased in resistance after blowing the compressed nitrogen gas over the samples with the healed microelectrodes.

It can be concluded from analysing the data of the healed microelectrodes exposed to various stress tests that PPy coverage does not offer additional protection, but it does decrease overall resistance of the healed microelectrodes.

## 4. Conclusions

The results presented in this study establish a proof of concept that electrically conductive CNT bridges can be assembled across large electrode gaps to heal the electrodes using dielectrophoretic step-wise deposition of carbon nanotubes from the suspension. The multiphysics simulation illustrated the key concept behind the introduced electrokinetic assembly process—the self-reinforcing nature of the DEP force experienced by the CNTs as the bridge forms across the electrode gap, decreasing the effective gap distance and increasing the magnitude of the applied electric field. The relationship between the resistance of the resulting CNT bridges and the length of the gaps they span was evaluated, which confirms the dominant role played by the CNT-to-CNT contact resistance in overall bridge resistance. The present study focused on the development of technology for healing of the conventional straight microelectrodes and, if extended to healing of the closely spaced and/or interdigitated electrodes close attention should be paid to the placement of the CNT suspension droplets to avoid unintended short circuits.

While further study of the proposed electrokinetic assembly process is needed to fully understand it, the ability to create long conductive CNT bridges is an exciting prospect with many potential applications. As research into microencapsulation of self-healing agents continues to develop [33,34,35,36,37], the self-reinforcing process presented in this paper offers impressive benefits for the use of CNTs in such microencapsulation systems. Conceptually, if CNT suspension is microencapsulated over the electrodes, then the guided process of CNT bridge creation presented in this work can be applied for self-healing of the electrodes by the inclusion of the regime to self-test electronic trances to locate the break and then apply the required AC bias to case step-wise deposition to take place. Additionally, the described dielectrophoretic step-wise fabrication of long conductive CNT bridges will find widespread application in many fields from nanoelectronics to tissue engineering.

## Figures and Tables

**Table 1:** Resistance measured across the electrode with 30 μm gap after deposition and drying of every two 0.5 μL droplets of CNT suspension.

Total Amount of CNT Suspension (μL)	Resistance (kΩ)	Total Amount of CNT Suspension (μL)	Resistance (kΩ)
1	open circuit	6	open circuit
2	open circuit	7	open circuit
3	open circuit	8	360
4	open circuit	9	147
5	open circuit	10	28.7

**Table 2:** Resistance of microelectrode before and after fracture and resistances of healed 20, 30, and 40 μm gap electrodes.

Electrode Gap Length (μm)	Avg. Resistance Measurement (Std. Dev.) (kΩ)
Microelectrode prior to fracture	5.80 (±0.61)
Microelectrode after fracture (>170 μm gap)	Open circuit
20	15.61 (±1.85)
30	19.11 (±1.88)
40	31.88 (±6.98)

**Table 3:** Resistance measurements before and after thermal cycling.

Sample	Resistance before Thermal Cycling (kΩ)	Resistance after Thermal Cycling (kΩ)
Without PPy Coating	28.7	23.2
With PPy Coating	0.93	1.09

**Table 4:** Resistance measurements before and after placing the healed electrodes under the deionised water (DI) water stream.

Sample	Resistance before Running DI Water over Healed Electrodes (kΩ)	Resistance after Running DI Water over Healed Electrodes (kΩ)
Without PPy Coating	23.0	24.8
With PPy Coating	1.41	1.50

**Table 5:** Resistance measurements before and after blowing compressed nitrogen over the samples of healed microelectrodes.

Sample	Resistance before Blowing Nitrogen over Healed Electrodes (kΩ)	Resistance after Blowing Nitrogen over Healed Electrodes (kΩ)
Without PPy Coating	23.4	23.0
With PPy Coating	1.26	1.24

## Data Availability

Data available upon request.
